# FLT3 mutations in canine acute lymphocytic leukemia

**DOI:** 10.1186/1471-2407-11-38

**Published:** 2011-01-27

**Authors:** Steven E Suter, George W Small, Eric L Seiser, Rachael Thomas, Matthew Breen, Kristy L Richards

**Affiliations:** 1 Department of Clinical Sciences, College of Veterinary Medicine, North Carolina State University, Raleigh, NC, USA; 2 Center for Comparative Medicine and Translational Research, North Carolina State University, Raleigh, NC, USA; 3 Lineberger Comprehensive Cancer Center, Chapel Hill, NC, USA; 4 Department of Molecular Biomedical Sciences, College of Veterinary Medicine, North Carolina State University, Raleigh, NC, USA; 5 Division of Hematology/Oncology, University of North Carolina at Chapel Hill; NC, USA

## Abstract

**Background:**

FMS-like tyrosine kinase 3 (FLT3) is a commonly mutated protein in a variety of human acute leukemias. Mutations leading to constitutively active FLT3, including internal tandem duplications of the juxtamembrane domain (ITD), result in continuous cellular proliferation, resistance to apoptotic cell death, and a poorer prognosis. A better understanding of the molecular consequences of FLT3 activation would allow improved therapeutic strategies in these patients. Canine lymphoproliferative diseases, including lymphoma and acute leukemias, share evolutionarily conserved chromosomal aberrations and exhibit conserved mutations within key oncogenes when compared to their human counterparts. A small percentage of canine acute lymphocytic leukemias (ALL) also exhibit *FLT3 *ITD mutations.

**Methods:**

We molecularly characterized *FLT3 *mutations in two dogs and one cell line, by DNA sequencing, gene expression analysis via quantitative real-time PCR, and sensitivity to the FLT3 inhibitor lestaurtinib via *in vitro *proliferation assays. FLT 3 and downstream mediators of FLT3 activation were assessed by Western blotting.

**Results:**

The canine B-cell leukemia cell line, GL-1, and neoplastic cells from 2/7 dogs diagnosed cytologically with ALL were found to have *FLT3 *ITD mutations and *FLT3 *mRNA up-regulation. Lestaurtinib, a small molecule FLT3 inhibitor, significantly inhibited the growth of GL-1 cells, while not affecting the growth of two other canine lymphoid cell lines without the *FLT3 *mutation. Finally, western blots were used to confirm the conserved downstream mediators of *FLT3 *activating mutations.

**Conclusions:**

These results show that ALL and FLT3 biology is conserved between canine and human patients, supporting the notion that canine ALL, in conjunction with the GL-1 cell line, will be useful in the development of a relevant large animal model to aid in the study of human FLT3 mutant leukemias.

## Background

FMS-like tyrosine kinase 3 (*FLT3*), one of the most commonly mutated genes in human leukemias, is a class III receptor tyrosine kinase that is an important regulatory gene involved in normal hematopoiesis [1,2]. FLT3 is expressed predominantly on myeloid and lymphoid hematopoietic progenitors, where the receptor, once bound by its cognate ligand (FLT3 ligand, FL), activates a variety of downstream targets. These include proteins in the signal transducers and activators of transcription (STAT), mitogen-activated protein (MAP) kinase, and AKT pathways that are all involved in regulating proliferation, differentiation, and cell survival [1,2]. *In vitro *studies have shown that constitutively activated FLT3 triggers downstream signaling pathways resulting in continuous cellular proliferation and resistance to apoptotic cell death. Constitutively activated FLT3 occurs via two main mechanisms: coexpression of FL, which leads to activation via autocrine, paracrine, or intracrine signaling, or via mutation of the *FLT3 *gene itself, conferring ligand independence [3-7]. Such mutations are internal tandem duplications of the juxtamembrane domain (ITD), point mutations of the juxtamembrane domain, or point mutations of the second tyrosine kinase domain (TKD). In transgenic murine model systems, constitutively activated FLT3 contributes to the leukemic phenotype [1,2,8,9].

The majority of human acute leukemias, including 100% of B-cell lineage acute lymphoblastic leukemias (ALL), 27% of T-lineage ALL, and 89% of acute myelogenous leukemias (AML) overexpress FLT3 [10,11]. ITD mutations are found in 3% of patients with myelodysplastic syndromes (MDS) [1,12], and up to 15% and 25% of pediatric and adult AML patients, respectively [1,2,13-15]. In both pediatric and adult AML patients, the presence of an ITD mutation is associated with a significantly higher relapse rate and worse overall survival [13-15]. *FLT3 *ITD mutations rarely occur in adult acute lymphoblastic leukemias (ALL) of B-cell origin and childhood ALL [1,2]. Interestingly, some of the highest levels of FLT3 expression occur in infant and childhood ALL, therefore, a mechanism other than mutation constitutively activates FLT3 in these cases [16]. As a therapeutic target, FLT3 is appealing since it is up-regulated in a significant number of acute leukemias and its protein expression is restricted to primitive and immature hematopoietic progenitors. Modest results from clinical trials with a variety of small-molecule FLT3 inhibitors suggest that improved understanding of *FLT3 *mutations and the resultant aberrations in signaling may be needed before we realize the full therapeutic potential of these agents.

The domestic dog (*Canis familiaris*) is a useful large-animal model of naturally occurring cancers, including hematologic malignancies such as lymphomas and leukemias. Canine hematologic malignancies share extensive similarities with their human counterparts with regards to clinical presentation, tumor biology and response to therapy [17,18] and, in addition, human and canine hematologic malignancies share evolutionarily conserved chromosomal aberrations as well as conserved mutations within key oncogenes [19,20]. Therefore, canine hematologic malignancies are recognized as appropriate models of their human counterparts [17], and comparative studies between human and canine patients may reveal common mechanisms of oncogenesis relevant to both species [21].

Recently, *FLT3 *mutations were reported in 4/57 (7%) of dogs with cytologically and immunophenotypically confirmed ALL [19], suggesting that this important mechanism of leukemia development and/or progression might be another instance of cross-species conservation of pathogenic mechanism. Three dogs with B-cell ALL harbored *FLT3 *ITD mutations of exons 14/15, while one dog with ALL of an unknown phenotype had a TKD-PM in exon 20. The functional consequences of the identified mutations (i.e. FLT3 up-regulation) were not examined.

Another suggestion of cross-species conservation of FLT3 activation came from our efforts to further characterize five previously reported malignant canine lymphoid cell lines- GL-1, CL-1, 17-71, CLGL-90, and CLL-1390 (manuscript submitted, Seiser EL, Thomas R, Richards KL, Byler KK, Breen T, Moore P, Suter SE, Breen M. Reading Between the Lines: Genomic Characterization of Five Widely Utilized Canine Lymphoid Tumor Cell Lines). Using array comparative genomic hybridization (aCGH) analysis, we found that GL-1 [22], a B-cell leukemia cell line, has an extensive copy number increase of the proximal half of dog chromosome 25, which harbors the *FLT3 *locus. Subsequent FISH analysis using a BAC clone containing the entire *FLT3 *sequence, showed a copy number increase to 4N on chromosome 25. These initial data suggested that the GL-1 cell line could be a relevant *in vitro *model for FLT3 activation, and if so, that canine leukemia models could be of use in the development of targeted FLT3 inhibitors.

The aim of this study was to determine if the canine GL-1 cell line and samples obtained from dogs diagnosed with ALL contain *FLT3 *mutations, to compare any identified mutations to those previously reported in both canine and human leukemia patients, and to determine whether the functional consequences of *FLT3 *mutation are conserved between dogs and humans.

## Methods

### Cell lines

The canine lymphoid cell lines, GL-1 (B-cell leukemia) [22], 17-71 (B-cell lymphoma) [23], and CLGL-90 (chronic large granular T cell leukemia, kind gift from Dr. Maxie Wellman, Ohio State University) [24], and human cell line, MV4-11 (biphenotypic myelomonocytic cell line) [25], were maintained in RPMI 1640 culture medium (Mediatech, Inc., Hendon, VA) supplemented with 10% fetal bovine serum (FBS, Mediatech, Inc), 2 mM L-glutamine (Mediatech, Inc) and 100 mg/ml Primocin (Invivogen, San Diego, CA) at 38°C/5% CO_2_. All cell lines tested negative for mycoplasma infection using a PCR-based test kit (AppliChem, Cheshire, CT). The GL-1 and CLGL-90 cell lines exhibit near normal ploidy (2n=78 for the domestic dog), with a range of 75-77 chromosomes and 77-82 chromosomes, respectively. The 17-71 cell line is hyperploid, with a range of 102-108 chromosomes. These cell lines were verified to match available published karyotypic and immunophenotyping information (manuscript submitted, Seiser EL, et al.).

### Sample recruitment

Three ml ethylenediaminetetraacetic acid (EDTA) blood samples were obtained during routine staging after owner consent via venipuncture from seven dogs presenting to the North Carolina State College of Veterinary Medicine Veterinary Teaching Hospital (NCSU-VTH) with clinical signs and hematologic abnormalities consistent with acute lymphoblastic leukemia. The study protocol was approved by the Institutional Animal Care and Use Committee of North Carolina State University. Blood smears stained with May Grunwald Giemsa were examined by a board-certified veterinary clinical pathologist. Morphological evaluation included a 200-cell differential white blood cell count. Flow cytometry was performed (Clinical Immunology Service, NCSU-VTH) using a panel of antibodies against CD3, CD4, CD8, CD21, CD45, CD34, CD14, and CD79a. A diagnosis of ALL was made based on a combination of morphological evaluation of blood and/or bone marrow smears, immunophenotyping, and hemogram analysis. These criteria included > 30% blasts in the peripheral blood or bone marrow, concurrent cytopenias, negative staining of the neoplastic cells using a myeloid marker (CD14), and positive staining using CD34, CD79a, CD21 (B-cell ALL), or CD34, CD3, CD4, and CD8 (T-cell ALL). ALL was differentiated from chronic lymphoid leukemia (CLL) and the leukemic phase of lymphoma on the basis of cell morphology, CD34 expression, and the distribution of organ involvement.

The canine ALL patients included two 8-year old and one 9-year old male castrated Labrador Retrievers, one 6-year old female spayed Schipperke, one 10-year old male castrated Border Collie, one 10.5-year old female spayed Doberman Pinscher, and one 8-year old male castrated mixed-breed dog. The ALL phenotypes were: three B-cell ALL, three ALL of unknown phenotype, and one dual-positive (i.e. T and B-cell markers) ALL. Five of these patients had ≥ 100,000/ml leukemic blasts in the peripheral blood (including both dogs harboring the *FLT3 *ITD), while three patients had < 30,000/ml leukemic blasts.

### Polymerase chain reaction (PCR)

Genomic DNA was prepared from whole blood collected by venipuncture from dogs diagnosed cytologically with ALL or from aliquots of cultured cell lines using the ZR Genomic DNA II kit (Zymo Research, Orange, CA) according to manufacturer instructions. PCR was conducted in 50 ul reactions containing 1 ul of genomic DNA (~35 ng); 25 ul Taq PCR MasterMix (Qiagen Inc., Valencia, CA), and 400 nM each of forward and reverse primers designed within intronic regions of the *FLT3 *gene and prepared by the Nucleic Acids Core Facility (University of North Carolina, Chapel Hill, NC). The FLT3 14/15 intronic primer pairs, 5'-CCA TTT CTG AGG GAC TGC-3' and 5'-GCC TTG AAA CAT GGC AAG C-3', were used for amplification of the exon regions 14 through 15 corresponding to the juxtamembrane domain [19]. The FLT3 20 intronic primer pairs, 5'-TCA CCT GGA ATT CCT ACT GAA C-3' and 5'-TGT ACT ACA GCG GTT GTG GAC-3' were used for amplification of exon 20 corresponding to the tyrosine kinase domain [19]. PCR reaction conditions were: 95°C for 3 min; followed by 37 cycles of 94°C for 30 s; 52°C (FLT3 14/15) or 55°C (FLT3 20) for 30 s; and 72°C for 1 min with a final elongation step of 72°C for 10 min. PCR amplicons were separated through a 1.5% agarose gel, followed by ethidium bromide staining and visualization under ultraviolet light. DNA was extracted from bands excised from the gel using a QIAquick Gel Extraction kit (Qiagen). Purified DNA was then sequenced using the previously mentioned PCR primers (Genomic Analysis Core Facility, University of North Carolina, Chapel Hill, NC).

### RNA preparation and quantitative real-time PCR

Peripheral blood mononuclear cells from a normal dog were isolated as previously described [26], then frozen in Trizol (Invitrogen, Carlsbad, CA) until RNA extraction per manufacturer's instructions. ALL case 1 had an excisional lymph node biopsy that was immediately stored in Trizol at -80°C until extraction. Cell line pellets were resuspended and frozen in Trizol at -80°C until extraction. RNA from other ALL cases was obtained from 200 ul of peripheral blood added to 1 ml RNAlater (Qiagen), then frozen at -20°C until Trizol extraction.

Total RNA from each sample was used to generate cDNA for downstream analysis (Quantitect Reverse Transcription Kit, Qiagen). Quantitative real-time PCR (qRT-PCR) was performed using the Quantitect SYBR Green Kit (Qiagen) and an iCycler (BioRad, Hercules, CA). The gene *RPL32 *was selected as the reference for quantitative analysis based on its stable expression across all samples examined by gene expression microarray (see below); PCR primers (forward: 5'-ATG CCC AAC ATT GGT TAT GG-3', reverse: 5'-CTC TTT CCA CGA TGG CTT TG-3') were designed as described previously [27]. *FLT3 *primers (Entrez GeneID: 486025, forward: 5'-CAG AGG CAG TGT ATG GAG CA-3', reverse: 5'-GGC AAT TCA GGG AAC TGT GT-3') were designed using NCBI Primer-BLAST (http://blast.ncbi.nlm.nih.gov/Blast.cgi). The reaction efficiency for the primer sets used was calculated using serial dilutions of cDNA, with efficiencies ranging from 90%-100%. Relative quantification was performed as described previously [28]. All qRT-PCR assays were performed in triplicate.

### Proliferation assays

Cells were seeded at 4 × 10^4 ^cells in a 96-well plate in a total volume of 200 ul and treated with 5 or 10 nM lestaurtinib for 96 hours. Proliferation was measured using Alamar Blue (AbD Serotec Ltd, Oxford, UK) according to manufacturer instructions. Fluorescence was measured using a DTX880 Multimode Detector (Beckman Coulter, Brea, CA) with excitation at 535 nm and emission at 595 nm. All assays were performed in triplicate and growth experiments were each repeated three times; standard error of the mean was calculated for the averages. Proliferation was expressed relative to untreated controls.

### Western blotting

Cells were seeded at 2 × 10^6 ^cells in 2 ml of RPMI supplemented with penicillin G sodium (100 U/ml), streptomycin sulfate (100 ug/ml), and 0.5% bovine serum albumin under serum-free conditions for 24 hours and then treated for 2 hours with or without 10 nM lestaurtinib (LC Laboratories, Woburn, MA). Cells were then lysed in RIPA buffer (Dulbecco's PBS, 0.1% Sodium Dodecyl Sulfate, 0.5% Sodium Deoxycholate, and 1% Ipegal NP-40 supplemented with 1 mM PMSF (phenylmethylsulfonylfluoride), 1x complete protease inhibitor cocktail (Roche Diagnostics, Indianapolis, IN), and 1x Phosphatase Inhibitor Cocktail 2 (Sigma, St. Louis, MO)). An aliquot was used for protein quantification using the BCA protein assay kit (Pierce Technology, Inc., St. Louis, MO). The remaining sample was mixed with 0.5 volumes of 3x SDS sample buffer (1x equals 31.25 mM Tris-HCl (pH 6.8), 2% SDS, 5% 2-mercaptoethanol, 10% sucrose, and 0.005% bromophenol blue). Equivalent protein amounts were run on SDS-PAGE in Tris/glycine/SDS buffer using 10% acrylamide gels. Resolved proteins were then transferred to a PVDF (polyvinylidene difluoride) membrane Antibodies directed against FLT3, phospho-FLT3, phospho-MAPK (Thr202/Tyr204) and phospho-Stat5 (Tyr694) (Cell Signaling, Boston, MA) were used. Anti-beta-actin (Sigma) was used as a loading control. Horseradish peroxidase-conjugated secondary antibodies were used with an ECL or ECL Plus detection reagent (GE Healthcare Bio-Sciences Corp., Piscataway, NJ) to visualize immunoreactive bands.

## Results

### Identification of *FLT3 *ITD mutations in a canine ALL cell line and in canine leukemia patient samples

**Figure 1:**
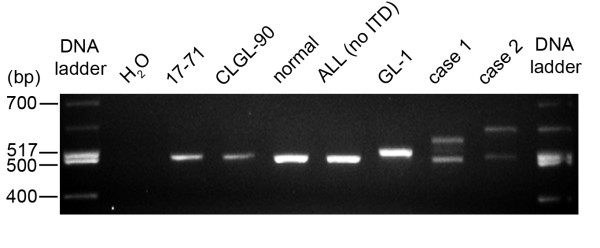
***FLT3 *PCR of genomic DNA**. *FLT3 *PCR with intronic primers bracketing exons 14 and 15 was performed and the products separated on an agarose gel. Samples containing a *FLT3 *ITD (GL-1, Case 1, and Case 2) have a band with decreased mobility compared to the wild-type *FLT3 *gene (500 bp in size), which is seen in 17-71, CLGL-90, a dog without ALL, and a representative dog with ALL without the *FLT3 *ITD.

We first evaluated the canine lymphoid cell lines for the presence of either exon 14/15 ITDs or exon 20 TKD point mutations using genomic PCR. Using previously published primers, the wild-type exon 14/15 product seen in the 17-71 and CLGL-90 cell lines is 500 bp (Figure 1, lanes 3, 4). In contrast, the GL-1 leukemia cell line yielded only a ~525 bp amplicon (Figure 1 lane 7), suggesting the possibility of an ITD and indicative of a complete loss of the wild-type *FLT3 *sequence. GL-1 cells, therefore, reveal loss of heterozygosity in the area of chromosome 25 containing the *FLT3 *gene (25 q LOH). No point mutations were identified by DNA sequencing of both exon 14/15 and exon 20 in any of the canine cell lines.

We next examined DNA isolated from seven dogs diagnosed with ALL. A wild-type 500 bp amplicon was produced when examining DNA isolated from a healthy dog and five dogs diagnosed with ALL (Figure 1, lanes 5, 6 and data not shown), and this was confirmed by DNA sequencing in each case. In contrast, *FLT3 *ITDs of differing sizes were found in two other dogs diagnosed with ALL (Figure 1, lanes 8, 9). In total, *FLT3 *ITDs were found in 2/7 (28.5%) dogs diagnosed with ALL. In contrast to the GL-1 ITD, which produced only one > 500 bp amplicon, the wild-type *FLT3 *allele was also seen in these canine ALL blood samples, either indicating heterozygosity or contamination with non-leukemic cells. Similar to the cell line analysis, no point mutations were found in exons 14/15 or exon 20 in any of the seven cases.

**Figure 2:**
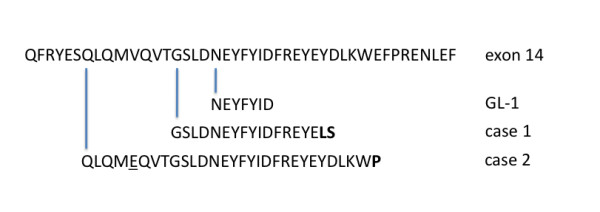
***FLT3 *ITD mutations in exon 14**. The amino acid sequences of the three *FLT3 *ITD mutations are shown, with the duplicated region shown below the wild-type sequence of exon 14 for each case. The bolded amino acid(s) are not part of the wild-type sequence and exist between the two repeats (not duplicated). The underlined residue in Case 2 is mutated in the duplication only; the other copy is wild-type. Each of the three ITD mutations are in-frame.

The predicted amino acid sequence of the ITDs is shown in Figure 2, based on our DNA sequence data. The GL-1 ITD is an in-frame 21 bp duplication, while the clinical cases contain in-frame 47 bp and 87 bp duplications, respectively. All maintain the same open reading frame as the original *FLT3 *sequence. Intervening bases between the repeats that maintain the reading frame in Cases 1 and 2 cause the insertion of an extra one to two bases between duplications (Figure 2). Case 2 has an additional point mutation resulting in a V to E change in the duplicated repeat (Figure 2).

### Overexpression of FLT3 in samples with a *FLT3 *ITD mutation

**Figure 3:**
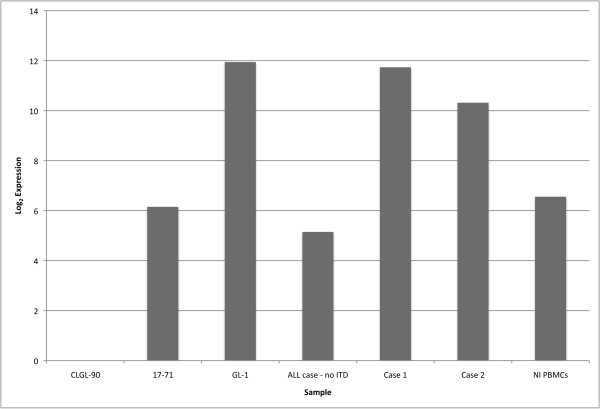
**Expression of *FLT3 *mRNA**. Samples were analyzed by quantitative PCR (Q-PCR) of *FLT3*. Expression levels relative to CLGL-90, which is normalized to zero, are shown in log_2 _units.

To begin to define the functional consequences of *FLT3 *mutations, we compared *FLT3 *mRNA expression between samples with and without ITDs. Figure 3 shows qRT-PCR results for the three canine lymphoid cell lines, normal canine peripheral blood, and canine ALL cases with and without *FLT3 *ITDs. GL-1 *FLT3 *mRNA expression was up-regulated relative to other lymphoid cell lines by more than 30-fold. Likewise, the two clinical ALL cases with a *FLT3 *ITD mutation had more than 30-fold increased expression relative to the ALL case without a *FLT3 *ITD mutation.

### Canine *FLT3 *ITD mutations are associated with lestaurtinib sensitivity

**Figure 4:**
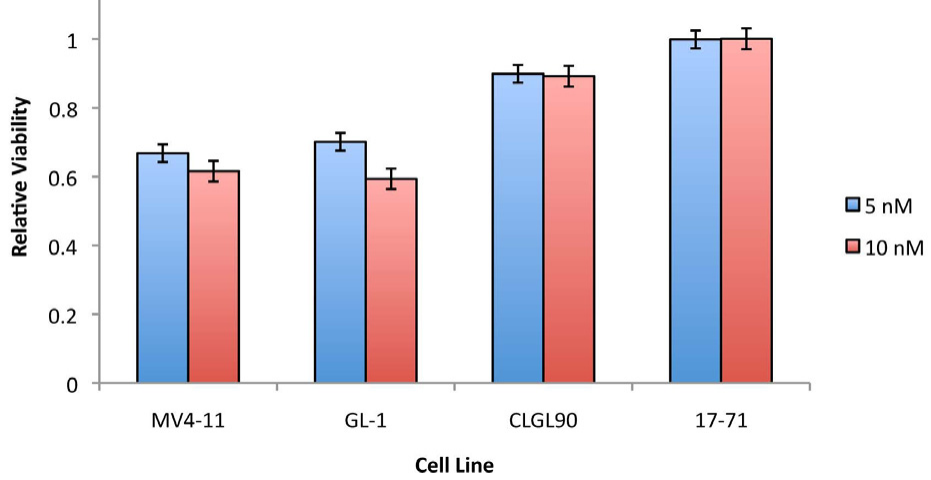
**Growth inhibition of *FLT3 *ITD-containing cell lines by lestaurtinib**. Cells were grown in 5 or 10 nM lestaurtinib. Viability of each cell line is shown relative to its growth in media without lestaurtinib. Experiments were done in triplicate, and standard error of the mean is shown.

The *FLT3 *ITD results in sensitivity to FLT3 inhibition in human cells *in vitro*. We determined whether the GL-1 cell line, containing the *FLT3 *ITD, is sensitive to the FLT3 tyrosine kinase inhibitor, lestaurtinib (Figure 4). A dose dependent growth inhibition of the GL-1 cells was clearly evident, with an approximately 40% growth reduction after being exposed to 10 nM lestaurtinib for 96 hrs. This was similar to the response of MV4-11, a human leukemia cell line with a *FLT3 *ITD mutation and documented sensitivity to lestaurtinib [29,30]. In contrast, the 17-71 cell line was essentially resistant to the growth inhibitory effects of lestaurtinib, while the CLGL-90 cell line showed only a modest reduction in growth. The culture conditions required to keep primary canine lymphoma and leukemia cells alive for relevant *in vitro *studies are not currently known, therefore, the growth inhibitory effect of lestaurtinib on neoplastic cells isolated from the canine ALL patients was not examined.

### Canine *FLT3 *ITD mutation activates the same downstream mediators as in humans

**Figure 5:**
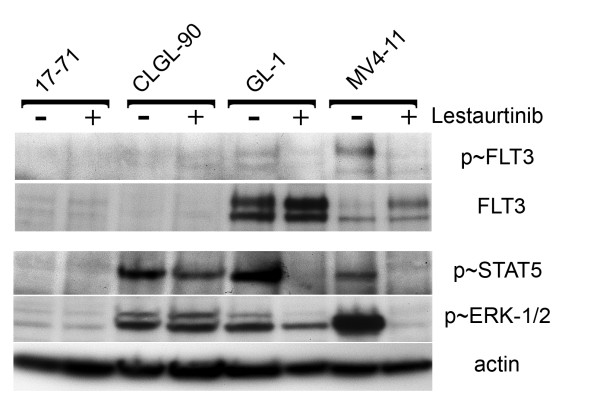
**Downstream mediators of *FLT3 *ITD**. Protein levels of FLT3, phospho-FLT3, phospho-STAT5 and phospho-ERK were assayed by Western blotting. Protein was harvested from cell lines that were grown both with and without lestaurtinib. Beta-actin was used as a loading control.

Finally, in an effort to determine if the downstream effectors of FLT3 inhibition are similar between the canine and human cell lines, we examined the expression and activation of FLT3 and members of the JAK/STAT and MAP kinase pathways with and without exposure to lestaurtinib (Figure 5). Upon FLT3 phosphorylation and dimerization, both downstream secondary mediators STAT5 and ERK1/2 are activated via phosphorylation of key residues, which leads to transit into the nucleus and increased transcription of a variety of genes involved in cell survival and differentiation. Furthermore, it has been reported that STAT5 activation is a specific effect of the *FLT3 *ITD, as opposed to other mechanisms of FLT3 up-regulation [31-33]. The canine cell line 17-71 expresses little to no phospho-STAT and phospho-ERK1/2, therefore, no effect of lestaurtinib on this cell line was seen using this assay. The CLGL-90 cell line, on the other hand, expressed a detectable amount of both STAT and ERK1/2. Growth in lestaurtinib had no effect on the phosphorylation status of these two proteins. In contrast, both the human MV4-11 and canine GL-1 cell lines showed a decrease in the phosphorylation of FLT3 (although the phospho-FLT3 antibody did not detect canine FLT3 as strongly as human FLT3), STAT5, and ERK1/2 when cultured in the presence of lestaurtinib. In addition, the level of phosphorylated STAT5 was undetectable after exposure to lestaurtinib, while the level of phosphorylated ERK1/2 was decreased in both cell lines.

## Discussion

We have demonstrated that both canine and human leukemias share *FLT3 *ITD mutations, in agreement with a previous report [19]. In addition, we show that a canine B-cell ALL cell line, GL-1, demonstrates amplification of the *FLT3 *ITD allele and loss of the wild-type *FLT3 *allele. Also, this is the first report of FLT3 overexpression in canine ALL clinical samples. Importantly, we also document the conserved functional consequences of the *FLT3 *ITD mutation. This includes constitutively activated FLT3, leading to increased phosphorylation of downstream mediators, and subsequent sensitivity to the FLT3 inhibitor, lestaurtinib.

Our previous high-resolution aCGH results showing amplification of the *FLT3 *locus in canine GL-1 cells led us to characterize this gene in more detail. Indeed, FLT3 expression in the GL-1 line is massively up-regulated, and the GL-1 cell line contains the *FLT3 *ITD while lacking wild-type *FLT3 *(Figure 1). In human AML, copy-neutral loss of wild-type *FLT3*, also referred to as acquired segmental uniparental disomy (aUPD), is associated with high-risk disease [34]. In a previous survey of canine leukemias, a heterozygous *FLT3 *ITD was found in 3/36 (~8.3%) dogs diagnosed with ALL [19]. Similar to our results, LOH of dog chromosome 25 q was not seen in this small number of clinical leukemia samples, therefore, loss of wild-type *FLT3 *is not common in clinical specimens. Although the GL-1 cell line does have 25 q LOH, the normal karyotype reported when this line was originally published cannot distinguish whether loss of the wild-type allele and/or amplification of the *FLT3 *ITD was present initially or developed with continued *in vitro *propagation. Regardless, the resulting genotype leads to extensive up-regulation of FLT3 in the GL-1 cell line.

Our results differ from human leukemia in that we observed the *FLT3 *ITD mutation not infrequently in ALL (2/7 samples), whereas in humans, the *FLT3 *ITD mutation is only rarely seen in ALL (~1% of ALLs) and is much more common in AML (25% of adult cases; 15% of pediatric cases) [1,2,13-15]. AML is exceedingly rare in dogs, while ALL is much more common. In canine patients, both diseases are characterized by an extremely aggressive biology with overall short survival times (weeks) when treated with standard multi-agent chemotherapy. Although *FLT3 *ITDs are uncommon in human ALL, FLT3 overexpression has been implicated in the pathogenesis of infant and childhood ALL [16]. Our results showing that *FLT3 *mutations lead to overexpression and/or activation of FLT3 in both the GL-1 cell line and clinical canine ALL cases is similar to findings examining human ALL cell lines and infant/childhood ALL, although the mechanism of activation more resembles that in human AML (i.e. ITD mutation).

In conjunction with others reports, our results show that, similar to human AML, canine ALL samples also contain differing lengths of ITDs. Also, the canine ITDs maintain the reading frame of the FLT3 protein. These similarities suggest that the mechanism of mutation is conserved across species. Data from human patients suggest that a longer ITD mutation is associated with lower CR rates and worse survivals [35,36]. Confirmation in dogs awaits larger numbers and improved treatments to increase overall survival times to a point where cross-species comparisons are possible.

FLT3 plays an important developmental role and is overexpressed in most patients with AML, therefore, it is an attractive target for the development of selective small molecule inhibitors. Lestaurtinib, a FLT3 inhibitor, selectively kills human ALL cell lines and primary childhood ALL cells that express high levels of FLT3. It is also more toxic to AML cell lines with a *FLT3 *ITD than those without it [29,30]. Our findings demonstrate that the canine GL-1 cell line, which exhibits high-level FLT3 expression and the *FLT3 *ITD mutation, is also sensitive to low concentrations of lestaurtinib. This result mirrors the effects of lestaurtinib on the human leukemia cell line MV4-11, which, like GL-1, contains a *FLT3 *ITD mutation with high expression levels [29,30]. In addition, we confirmed that the inhibition of proliferation of the GL-1 cell line by lestaurtinib is most likely mediated via decreased levels of the STAT5 and ERK1/2 phosphoproteins, all of which reinforces the underlying conservation between dysregulated pathways in humans and canine leukemia, even across diseases (AML and ALL, respectively).

## Conclusions

In summary, we present data that shows ALL/FLT3 biology is conserved across human and canine species. Canine ALL patients, in addition to the canine B-cell leukemia cell line, GL-1, contain *FLT3 *mutations that lead to FLT3 up-regulation and subsequent activation of members of the JAK/STAT and MAP kinase pathways. In particular, the GL-1 cell line, which has massively up-regulated FLT3 expression as well as a typical *FLT3 *ITD mutation, will serve as a sensitive tool to test FLT3 selective inhibitors. Thus, this data will lay the groundwork for future studies of leukemogenesis, supporting the notion that canine ALL can be viewed as a relevant large animal model for human *FLT3 *ITD-positive leukemias and is useful for the development of novel therapeutics and/or treatment strategies for both dogs and humans.

## Competing interests

KLR was on the speakers bureau for Cephalon Oncology in 2008-2009.

## Authors' contributions

SES contributed to the conception and design of the study, collected the clinical samples, analyzed data and wrote the manuscript. GWS performed the *FLT3 *PCR and mutation analysis, the FLT3 western blots, the cell culture experiments, and analyzed data. ELS performed the *FLT3 *expression experiment and analyzed data. RT and MB analyzed data and edited the manuscript. KLR contributed to the conception and design of the study, analyzed data, and revised the manuscript. All authors read and approved the final manuscript.

## Pre-publication history

The pre-publication history for this paper can be accessed here:

http://www.biomedcentral.com/1471-2407/11/38/prepub
